# Supplementary material for: Prevalence Rates of Depression and Anxiety among Young Rural and Urban Australians: A Systematic Review and Meta-Analysis
Source: Int J Environ Res Public Health. 2023 Jan 1;20(1):800. doi: 10.3390/ijerph20010800 (PMC9819515; doi:10.3390/ijerph20010800)
Supplement: Supplementary file 1 [file ijerph-20-00800-s001.zip › ijerph-2104063-supplementary.pdf]

Supplementary Material

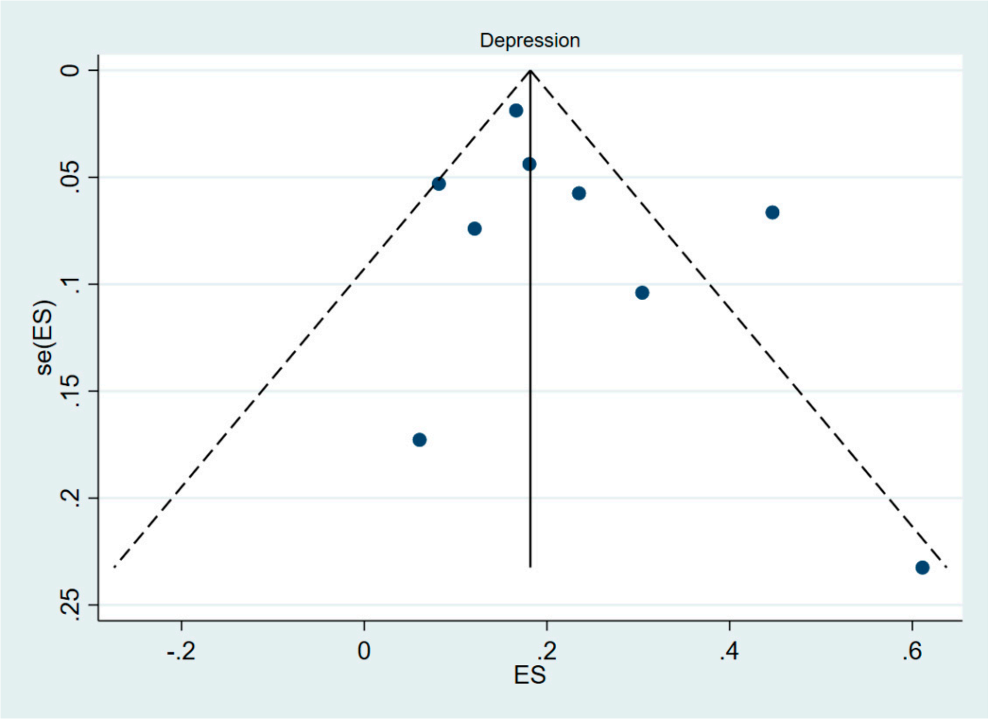

Figure S1. Begg's funnel plot: depression.

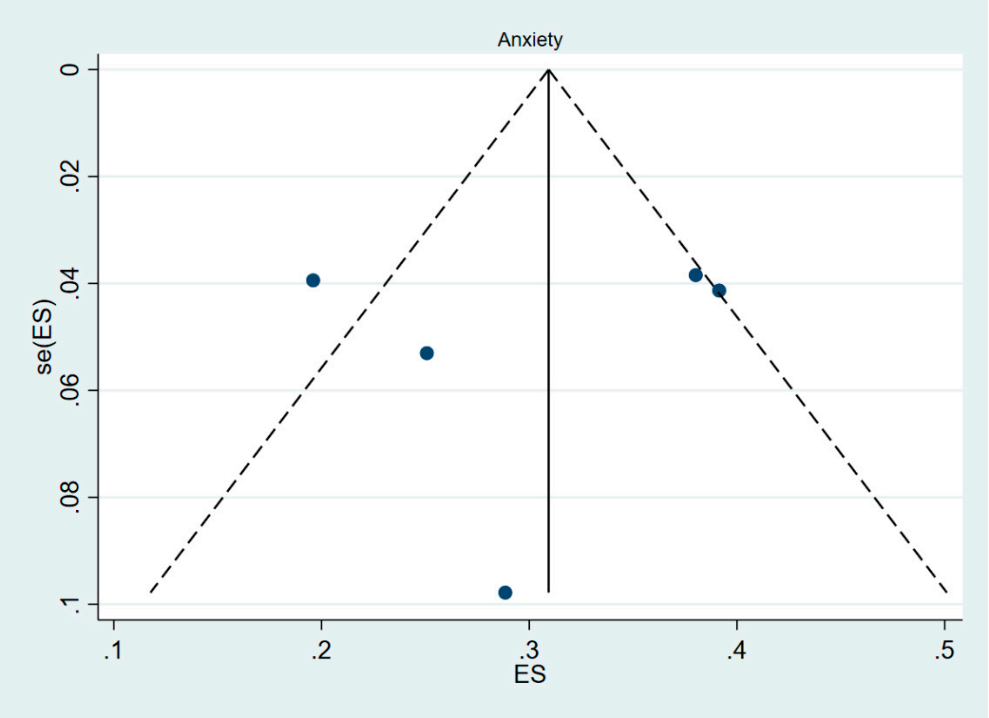

Figure S2. Begg's funnel plot: anxiety.

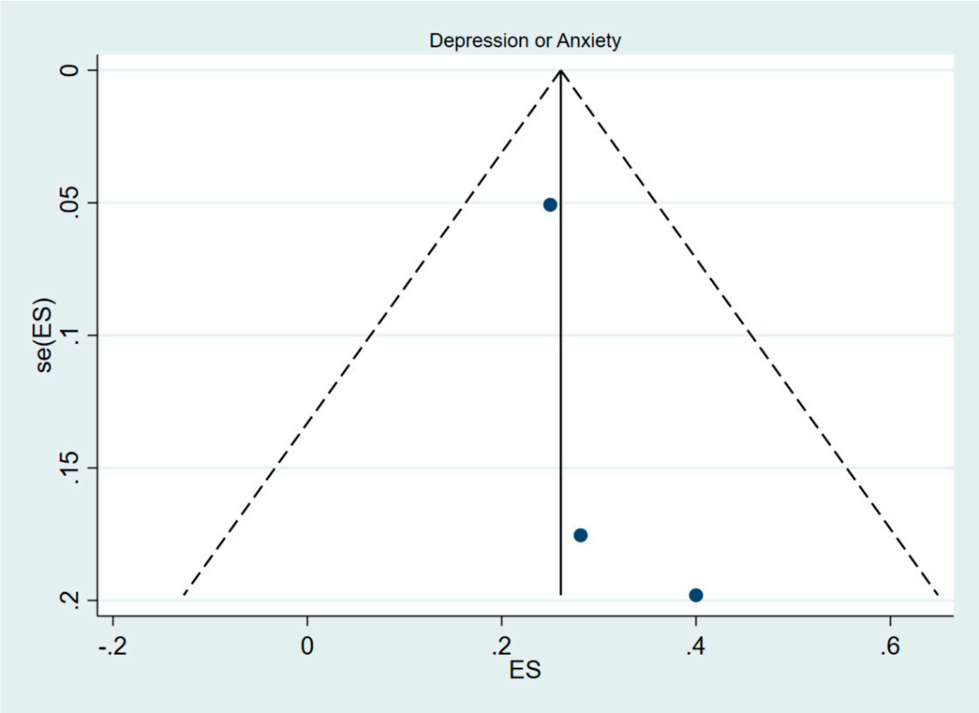

Figure S3. Begg's funnel plot: depression or anxiety.

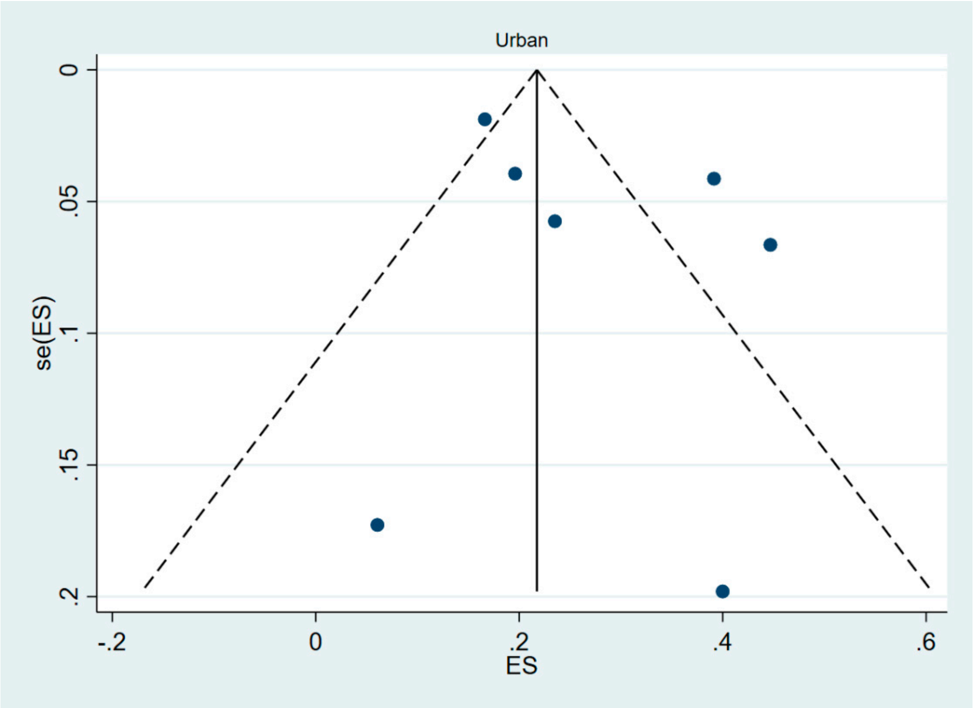

Figure S4. Begg's funnel plot: urban.

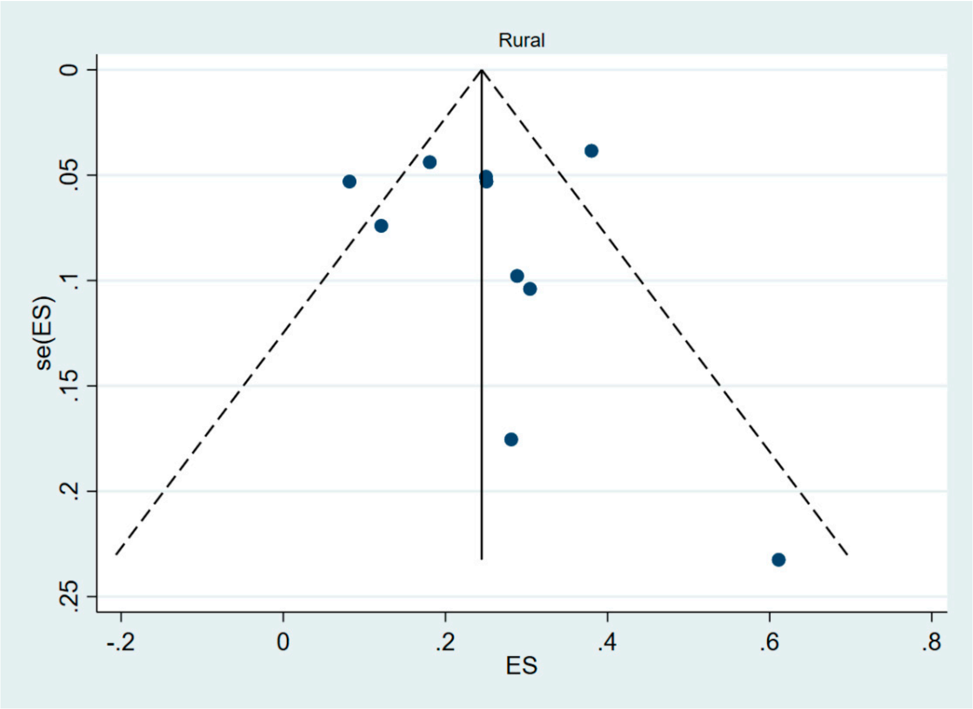

Figure S5. Begg's funnel plot: rural.

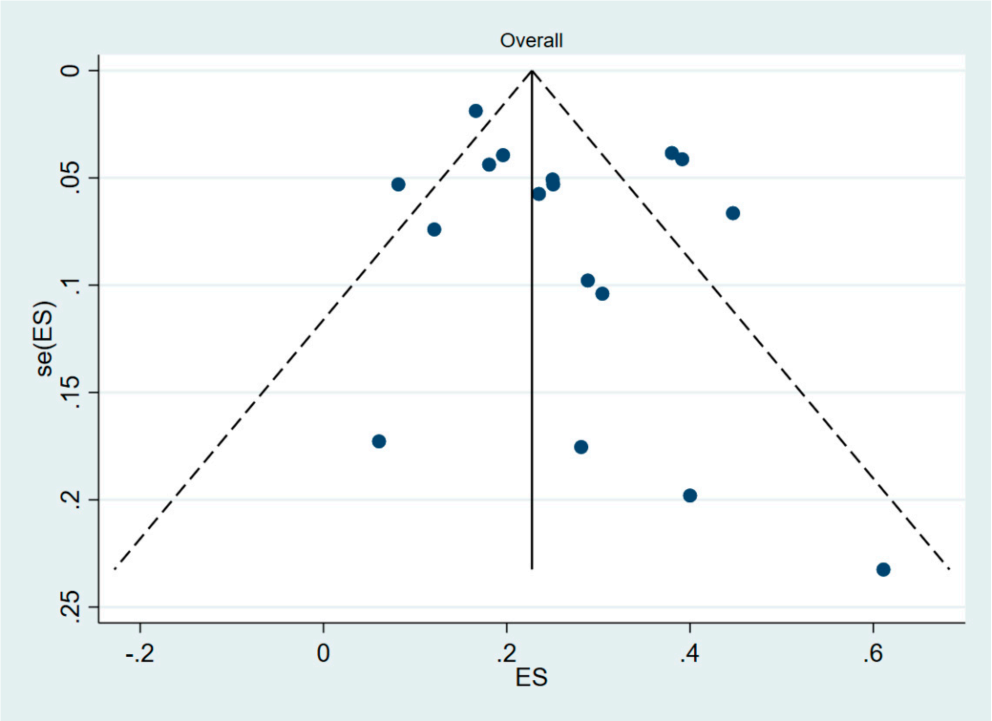

Figure S6. Begg's funnel plot: overall.

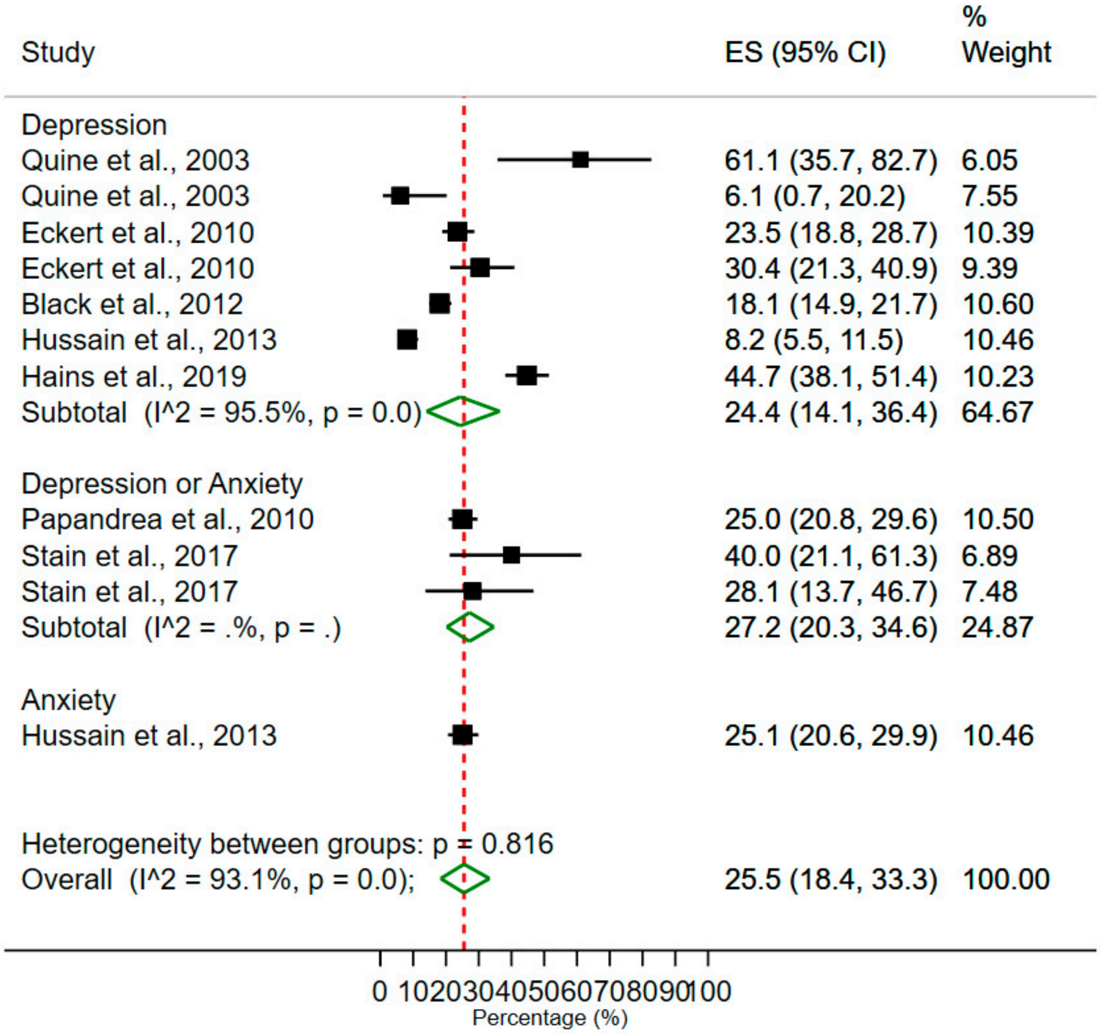

Figure S7. Sensitivity analysis: pooled estimate of depression, anxiety and depression or anxiety disorders without studies of wide range of age groups [1,4,5,6,7,8,9].

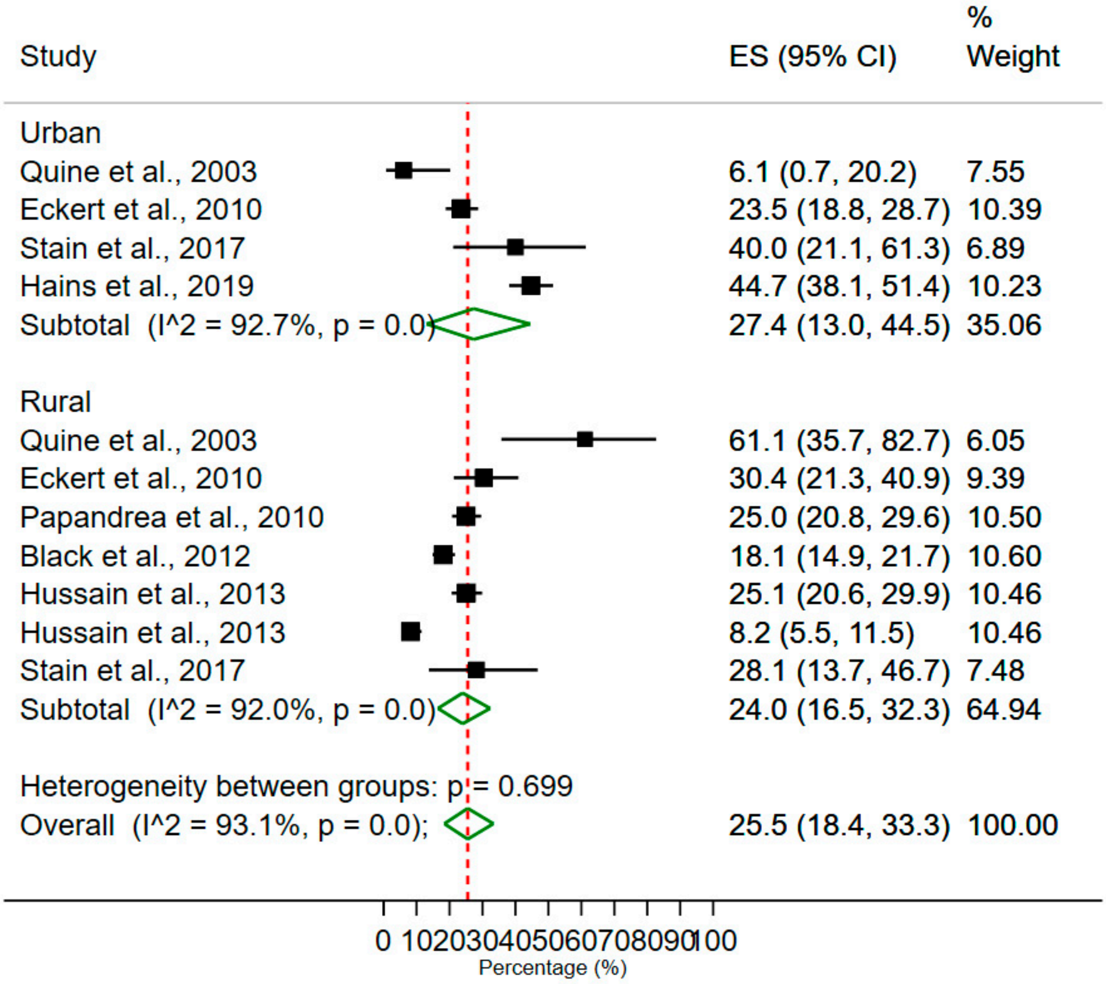

Figure S8. Sensitivity analysis: pooled estimate of urban area and rural areas without studies of wide range of age groups [1,4,5,6,7,8,9].

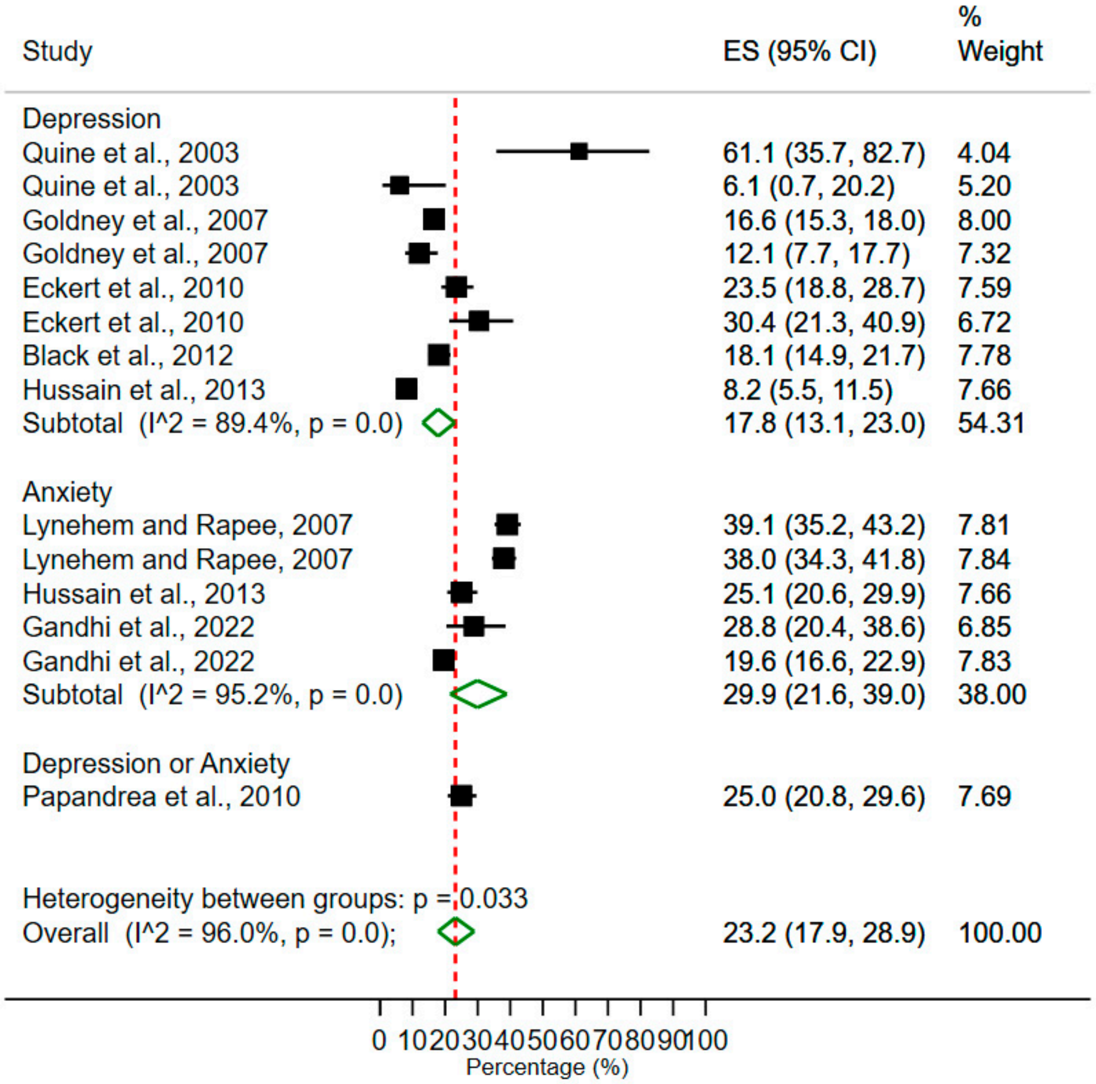

Figure S9. Sensitivity analysis: pooled estimate of depression, anxiety and depression or anxiety disorders without studies of "at-risk" participants [1,2,3,4,5,7,9,10].

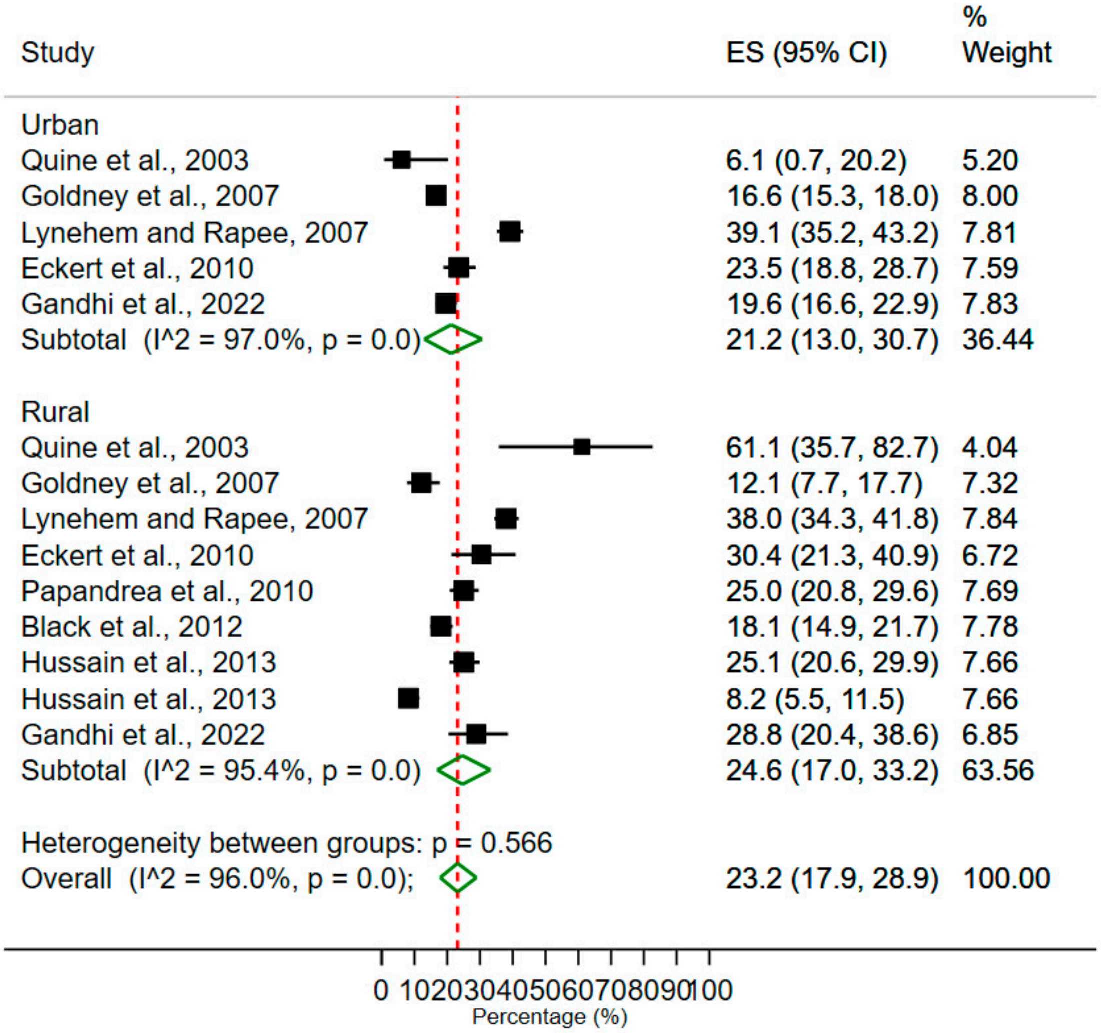

Figure S10. Sensitivity analysis: pooled estimate of urban area and rural areas without studies of "at-risk" participants [1,2,3,4,5,7,9,10].

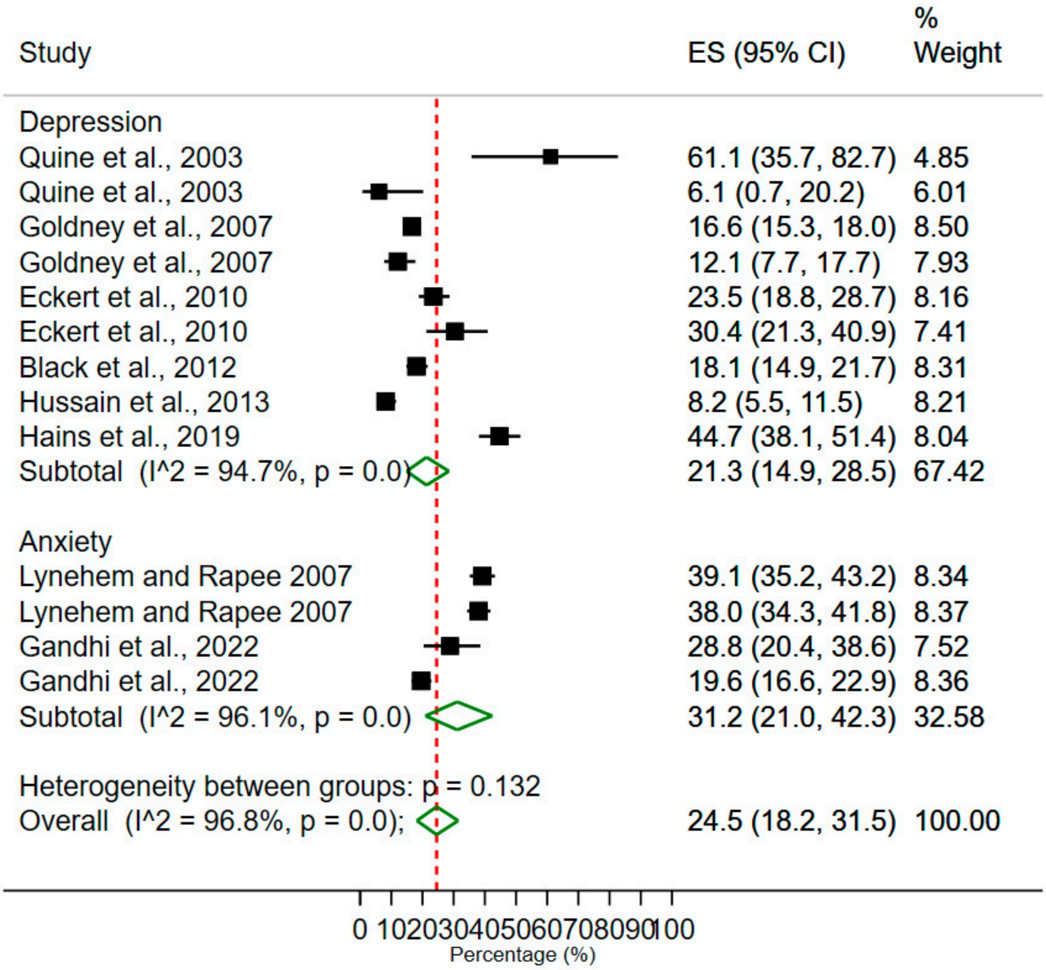

Figure S11. Sensitivity analysis: pooled estimate of depression, anxiety and depression or anxiety disorders without studies of that illustrated insignificant results [1,2,3,4,5,8,9,10].

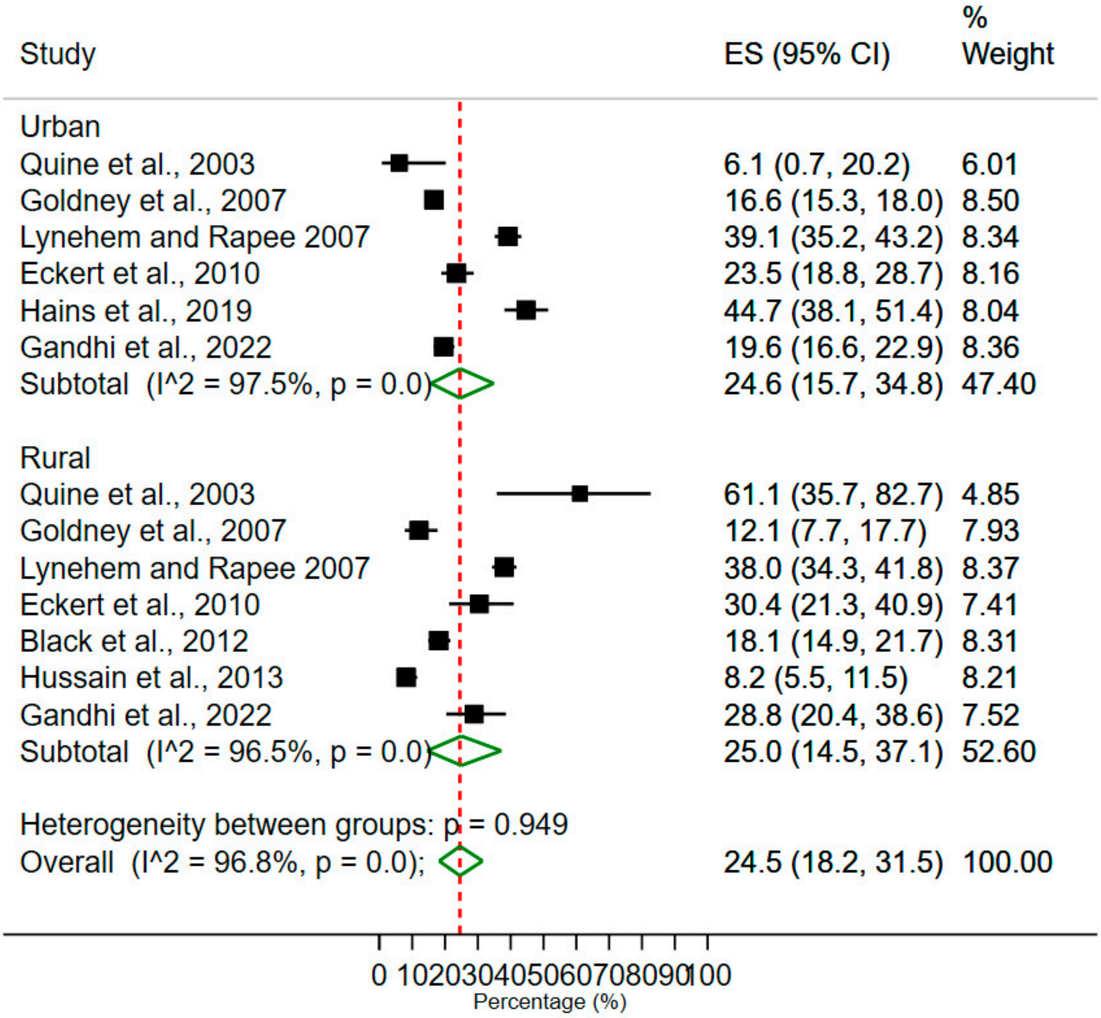

Figure S12. Sensitivity analysis: pooled estimate of urban area and rural areas without studies of that illustrated insignificant results [1,2,3,4,5,8,9,10].

```
Pooling
  Model: Random-effects
 Method: DerSimonian-Laird
```

Studies	Prevalence	[95% conf.	interval]
Observed	0.259	0.193	0.325
Observed + Imputed	0.253	0.187	0.318

Figure S13. Trim and Fill analysis: one imputed study.

Table S1. Base characteristics of the 17 estimates from the 10 studies included in the meta-analysis.

Title	Author	Year	Age	Disorder	Region	Prevalence Rate	Numerator	Denominator
Changes in depression-related mental health literacy in young men from Rural and urban South Australia	[1]	2010	15–30	Depression	Urban	23.50%	71	302
Changes in depression-related mental health literacy in young men from Rural and urban South Australia	[1]	2010	15–30	Depression	Rural	30.40%	28	92
Childhood anxiety in Rural and urban areas: Presentation, impact and help seeking*	[2]	2007	5–12	Anxiety	Urban	39%	229	585
Childhood anxiety in Rural and urban areas: Presentation, impact and help seeking*	[2]	2007	5–12	Anxiety	Rural	38%	257	676
Depression and remoteness from health services in South Australia	[3]	2007	15–85	Depression	Urban	16.80%	471	2833
Depression and remoteness from health services in South Australia	[3]	2007	15–85	Depression	Rural	12.10%	22	182
Depression in Rural adolescents: relationships with gender and availability of mental health services	[4]	2012	13–18	Depression	Rural	18%	94	520
Health and access issues among Australian adolescents: a Rural-urban comparison	[5]	2003	12–17	Depression	Rural	61.10%	11	18
Health and access issues among Australian adolescents: a Rural-urban comparison	[5]	2003	12–17	Depression	Urban	6.06%	2	33
Impact of Rurality and substance use on young people at ultra-high risk for psychosis	[6]	2017	12–25	Both	Urban	40.00%	10	25
Impact of Rurality and substance use on young people at ultra-high risk for psychosis	[6]	2017	12–25	Both	Rural	28.10%	9	32
Oiling a neglected wheel: An investigation of adolescent internalising problems in Rural South Australia	[7]	2010	13–15	Both	Rural	25%	97	388
Perceived Burdensomeness Predicts Outcomes of Short-Term Psychological Treatment of Young People at Risk of Suicide	[8]	2019	12–25	Depression	Urban	44.70%	101	226
Physical and mental health perspectives of first year undergraduate Rural university students	[9]	2013	20	Anxiety	Rural	25%	89	355
Physical and mental health perspectives of first year undergraduate Rural university students	[9]	2013	20	Depression	Rural	8%	29	355
Receipt of evidence-based care for children and adolescents with anxiety in Australia	[10]	2022	4–18	Anxiety	Rural	28.85%	30	104
Receipt of evidence-based care for children and adolescents with anxiety in Australia	[10]	2022	4–18	Anxiety	Urban	19.6%	126	643

References

1. Eckert, K.A.; Kutek, S.M.; Dunn, K.I.; Air, T.M.; Goldney, R.D. Changes in depression-related mental health literacy in young men from rural and urban South Australia. *Aust. J. Rural Health* **2010**, *18*, 153–158. https://doi.org/10.1111/j.1440-1584.2010.01135.x.
2. Lynehem, J.H.; Rapee, M.R. Childhood anxiety in rural and urban areas: Presentation, impact and help seeking. *Aust. J. Psychol.* **2007**, *59*, 108–118. https://doi.org/10.1080/00049530701317082.
3. Goldney, R.D.; Taylor, A.W.; Bain, M.A. Depression and remoteness from health services in South Australia. *Aust. J. Rural Health* **2007**, *15*, 201–210. https://doi.org/10.1111/j.1440-1584.2007.00885.x.
4. Black, G.; Roberts, R.M.; Li-Leng, T. Depression in rural adolescents: Relationships with gender and availability of mental health services. *Rural Remote Health* **2012**, *12*, 2092. https://doi.org/10.22605/RRH2092.
5. Quine, S.; Bernard, D.; Booth, M.; Kang, M.; Usherwood, T.; Alperstein, G.; Bennett, D. Health and access issues among Australian adolescents: A rural-urban comparison. *Rural Remote Health* **2003**, *3*, 245. http://dx.doi.org/10.22605/RRH245.
6. Stain, H.J.; Halpin, S.A.; Baker, A.L.; Startup, M.; Carr, V.J.; Schall, U.; Crittenden, K.; Clark, V.; Lewin, T.J.; Bucci, S. Impact of rurality and substance use on young people at ultra-high risk for psychosis. *Early Interv. Psychiatry* **2018**, *12*, 1173–1180. https://doi.org/10.1111/eip.12437.
7. Papandrea, K.; Winefield, H.; Livingstone, A. Oiling a neglected wheel: An investigation of adolescent internalising problems in rural South Australia. *Rural Remote Health* **2010**, *10*, 1524. https://doi.org/10.22605/RRH1524.
8. Hains, A.; Janackovski, A.; Deane, F.P.; Rankin, K. Perceived burdensomeness predicts outcomes of short-term psychological treatment of young people at risk of suicide. *Suicide Life-Threat. Behav.* **2019**, *49*, 586–597. https://doi.org/10.1111/sltb.12452.
9. Hussain, R.; Guppy, M.; Robertson, S.; Temple, E. Physical and mental health perspectives of first year undergraduate rural university students. *BMC Public Health* **2013**, *13*, 848. https://doi.org/10.1186/1471-2458-13-848.
10. Gandhi, E.; OGradey-Lee, M.; Jones, A.; Hudson, J.L. Receipt for evidence-based care for children and adolescents with anxiety in Australia. *Aust. N. Z. J. Psychiatry* **2022**, *56*, 1463–1476. https://doi.org/10.1177/00048674211068780.

Do-file for the results:

Meta analysis:

```
*clear any existing commands or data

clear

*import the data from the file path and set the first row as variable names

import excel "C:\Users\s438549\Documents\PhD\Systematic review\Meta analysis\Data.xlsx",
sheet("data") firstrow

*install meta analysis tool

ssc install metaprop

*install funnel plot to study risk bias

ssc install metafunnel

*install network analsysis package to use scheme function in the later steps

net install nwcommands-ado, from(http://www.nwcommands.org)

*run the meta analysis for disorder through random effects model, Freeman-Turkey double arcsine trans-
formation of proportion and exact confidence intervals.Set the graph specifications

metaprop Numerator Denominator, random ftt by(Disorder) cimethod(exact) label(namevar= Author) ///
        sortby( Year Author ) xlab(0, 10, 20, 30, 40, 50, 60, 70, 80, 90, 100) xtitle("Percentage (%)",size(2)) ///
    olineopt(lcolor(red) lpattern(shortdash))  diamopt(lcolor(green)) pointopt(msymbol(x)msize(0)) ///
    boxopt(msymbol(S) mcolor(black)) astext(70) texts(100) power(2) dp(1) scheme(s1network) name(Disor-
der, replace)


*run the meta analysis for region through random effects model, Freeman-Turkey double arcsine transfor-
mation of proportion and exact confidence intervals. Set the graph specifications

metaprop Numerator Denominator, random ftt by(Region) cimethod(exact) label(namevar= Author) ///
    sortby( Year Author ) xlab(0, 10, 20, 30, 40, 50, 60, 70, 80, 90, 100) xtitle("Percentage (%)",size(2)) ///
    olineopt(lcolor(red) lpattern(shortdash))  diamopt(lcolor(green)) pointopt(msymbol(x)msize(0)) ///
    boxopt(msymbol(S) mcolor(black)) astext(70) texts(100) power(2) dp(1) scheme(s1network) name(Re-
gion, replace)


*Funnel plots

metafunnel _ES _seES, subtit("Overall", size(small)) name(fun_tot, replace)
```

metafunnel _ES _seES if Region== "Urban", subtit("Urban", size(small)) name(fun_urb, replace)

metafunnel _ES _seES if Region== "Rural", subtit("Rural", size(small)) name(fun_rur, replace)

metafunnel _ES _seES if Disorder== "Depression", subtit("Depression", size(small)) name(fun_dep, replace)

metafunnel _ES _seES if Disorder== "Anxiety", subtit("Anxiety", size(small)) name(fun_anx, replace)

metafunnel _ES _seES if Disorder== "Depression or Anxiety", subtit("Depression or Anxiety", size(small)) name(fun_both, replace)

*View them all together

gr combine fun_rur fun_urb fun_dep fun_anx fun_tot, imargin(0)

*Install metabias for egger test

ssc install metabias

*run egger test

metabias _ES _seES, egger graph scheme(s1network)
